# The National White Cross Association

**Published:** 1912-02

**Authors:** 


### The National White Cross Association.

We are in receipt of Bulletin No. 6, "for professional Co-operation solely," and learn that the "Association was founded some years ago by doctors to obtain through common study and experiment, the best practical scientific aids for use in their practice." The insignia consists of a white Maltese cross bearing the device, at times of a girl with spinal curvature, at others of a stork with the inevitable baby. There follows an illustrated dissertation on spinal curvature, etc., hernia, varicose veins, corsets, anatomy, sphygmomanometers, vibrators, speaking tubes for deaf persons, whirling sprays—particularly appropriate to the stork insignia—high frequency coils, therapeutic lamps, pocket surgical sets, stethoscopes, pessaries, stationery, etc., with prices.

Unfortunately we—and the pronoun may be taken professionally or editorially—earn our living by the misfortunes of the people so that it may be a case of the pot calling the kettle black but, gentlemen of the exceedingly miscellaneous firms who are issuing this circular, we want to say just a few words in regard to advertising. We are not a sentimental profession or people but there are some things, like the cross and the flag, that are sacred. Aside from its ultimate religious symbolism, the cross, of whatever type and color, is accorded by common consent to various altruistic and philanthropic associations. It is not appropriate to business ventures, however legitimate these may be in themselves, any more than the temple was considered the appropriate location for money-changers by the One with whom the cross is especially associated.

There is another point worth considering. We confess that we were fooled by your announcement to the extent that we were prepared to donate time and printers' charges to whatever phase of philanthropy the Association might foster, whether it dealt with the "white plague," or the sexual evil, which naturally suggested themselves, or to any other good cause. But it is still a long time to the first of April and, even on that day, it is not good business to play too many jokes on prospective customers.

The form of your advertisement shows that you realize that a circular is not read by busy men, if they know it is an advertisement. Advertising is a perfectly legitimate phase of business, its value depends upon its legitimacy, just as truly as the ultimate market for any commodity depends upon its merit.

Finally, magazine advertising, is the cheapest in actual cost and the most efficient.

